# Draft Genome Sequence of *Chromobacterium vaccinii*, a Potential Biocontrol Agent against Mosquito (*Aedes aegypti*) Larvae

**DOI:** 10.1128/genomeA.00477-15

**Published:** 2015-05-21

**Authors:** Kristin Vöing, Alisha Harrison, Scott D. Soby

**Affiliations:** a Fachbereich Biologie, Universität Münster, Münster, Germany; b Biomedical Sciences Program, College of Health Sciences and College of Veterinary Medicine, Midwestern University, Glendale, Arizona, USA

## Abstract

*Chromobacterium vaccinii* has been isolated only from cranberry bogs in Massachusetts. While it is unknown what role these bacteria play in their natural environments, they hold potential as biological control agents against the larvae of insect pests. Potential virulence genes were identified, including the violacein synthesis pathway, siderophores, and chitinases.

## GENOME ANNOUNCEMENT

*Chromobacterium vaccinii* strains were isolated from cultivated and wild cranberry bog soils in Massachusetts (1). *C. vaccinii* is characterized by its production of the pigment violacein and its ability to kill diamondback moth and *Aedes* mosquito larvae (2). The genomes of isolates MWU205^T^ and MWU328 were sequenced at the University of Arizona Genetics Core using the 454 GS FLX Titanium system following the manufacturer’s protocols. Libraries were generated using the NEBNext Quick DNA library prep master mix set for 454, and MID-tagged using the GS FLX Titanium rapid library MID adaptors kit. The average size of randomly sheared DNA was 791 bp with a peak at 1,534 bp for MWU205 and 802 bp with a peak at 1,771 bp for MWU328. The emPCR Amplification Method Manual–Lib-L LV, XL+ (3) was followed for bulk emulsion-based clonal amplification PCR (emPCR) of each sample. Three samples were loaded into each region of a sequencing PTP plate divided with a two-region gasket. Roughly 670,000 beads were loaded per sample. Roche Newbler software version 2.9 was used for signal processing, sample demultiplexing, and partial assembly. The number of reads per sample for MWU205 was 230,669, giving coverage of 28×, and for MWU328 was 253,866, giving coverage of 31×. The resulting assembly of MWU205 included 204,417 (88.62%) scaffolds, consisting of 4,967,512 nt on 152 large scaffolds with an *N*_50_ of 79,933 nt and a maximum scaffold length of 215,390 nt. Assembly of MWU328 included 200,099 (91.17%) scaffolds, consisting of 4,958,868 nt on 123 large scaffolds with an *N*_50_ of 75,578 nt and a maximum scaffold length of 215,917 nt. MWU205 and MWU328 total genomes were compared using the Genome-to-Genome Distance Calculator (GGDC) provided online by the DSMZ. GGDC mimics *in vitro* DNA-DNA hybridization by dividing scaffold sequences into fragments approximately the same size as would be expected *in vitro*, and pairing up homologous segments (4–6). The MWU205 and MWU328 total genomes were found to be 88.1% homologous by this method and only 40% homologous to the type species/isolate of the genus *C. violaceum* ATCC 12472. The GC fractions of MWU205 and MWU328 are 0.64351 and 0.64292, respectively.

*Ab initio* gene prediction was performed on the assembly using RAST (http://rast.nmpdr.org). A number of potential virulence factors were observed that may contribute to larval toxicity, including production of the pigment violacein, siderophores, hydrogen cyanide, and secreted chitinases (7). MWU205 contained 36 probable chitinase genes, including 12 probable chitinase A genes, 6 endochitinases, and 21 hydrolase transmembrane family proteins. MWU328 contained 15 probable chitinase genes, including 66 probable chitinase A genes, 33 endochitinases, and 70 hydrolase transmembrane family proteins. The roles of chitinases, as well as those of other putative insect larvae virulence factors, are relatively unexplored in *C. vaccinii* and require further investigation.

### Nucleotide sequence accession numbers.

The whole-genome shotgun project for MWU205 has been deposited at DDBJ/EMBL/GenBank under the accession number JZJL00000000; the version described in this paper is JZJL01000000. The whole-genome shotgun project for MWU328 has been deposited at DDBJ/EMBL/GenBank under the accession number JZJJ00000000; the version described in this paper is JZJJ01000000.
